# Utilization and Costs of Mobile Medical Units for Veterans Experiencing Homelessness

**DOI:** 10.1001/jamanetworkopen.2025.55068

**Published:** 2026-01-30

**Authors:** Jean Yoon, Adam Chow, Jillian J. Weber, Emily P. Wong, Daniel M. Blonigen, Jack Tsai

**Affiliations:** 1 Health Economics Resource Center, Veterans Affairs (VA) Palo Alto Health Care System, Menlo Park, California; 2 Center for Innovation to Implementation, VA Palo Alto Health Care System, Menlo Park, California; 3 Department of Medicine, University of California, San Francisco; 4 Veterans Health Administration Homeless Programs Office, Department of Veterans Affairs, Washington, DC; 5 Department of Psychiatry and Behavioral Sciences, Stanford University School of Medicine, Stanford, California; 6 School of Public Health, University of Texas Health Science Center at Houston, Houston

## Abstract

**Importance:**

The Veterans Affairs (VA) health care system Homeless Patient Aligned Care Teams (HPACT) program began staggered deployment of mobile medical units (MMUs) to 24 VA medical centers in late 2023 to increase outreach and health care access in community settings for veterans experiencing homelessness.

**Objectives:**

To examine the early association between MMU implementation and the use of outpatient and inpatient services and costs among veterans experiencing homelessness.

**Design, Setting, and Participants:**

This longitudinal cohort study was performed at 24 HPACT sites from October 1, 2021, to March 30, 2025. Participants included veterans experiencing homelessness who used MMU care. Changes in utilization and costs in each fiscal quarter before and after site MMU implementation were estimated for patients treated by MMU relative to those receiving usual care.

**Exposure:**

MMUs providing primary care and other services in community settings.

**Main Outcomes and Measures:**

VA outpatient visits by type of care, VA inpatient stays by type of care, and VA outpatient, inpatient, and total costs of care, analyzed using linear difference-in-differences and event study models. Sensitivity analyses included a propensity score–matched comparison group and comparisons of all veterans experiencing homelessness in MMU sites and other HPACT sites.

**Results:**

A total of 2700 patients who used MMU care were included in the analysis (mean [SD] age, 60.5 [12.6] years). Of these, 2523 patients (93%) were male and had a mean (SD) Elixhauser comorbidity score of 3.3 (2.8). Per fiscal quarter, patients using MMU care had increases in mean number of visits for primary care (0.43; 95% CI, 0.30-0.56), homeless programs (1.40; 95% CI, 1.05-1.76), mental health intensive case management (0.03; 95% CI, 0.01-0.06), and emergency department care (0.06; 95% CI, 0.03-0.10); decreased mean inpatient stays for substance use disorders (−0.002; 95% CI, −0.004 to −0.0004) and domiciliary care (−0.002; 95% CI, −0.004 to −0.001); and increased mean total costs ($1724; 95% CI, $1172-$2276) compared with those receiving usual care after MMU implementation.

**Conclusions and Relevance:**

In this cohort study of veterans experiencing homelessness, patients receiving MMU care had small increases in primary care, mental health intensive case management, emergency department, and housing and other homeless services and few decreases in inpatient care at higher health care costs. MMUs may not reduce all types of acute care, but they may be an effective modality to reach veterans experiencing homelessness with access barriers and greater complexity and needs for care.

## Key Points

**Question:**

How is implementation of mobile medical units (MMUs) associated with health care utilization and costs for veterans experiencing homelessness?

**Findings:**

In this cohort study of 2700 participants, compared with usual care, implementation of MMUs was associated with increased utilization of primary care and mental health intensive case management and decreased use of inpatient substance use disorder and domiciliary care. MMUs were also associated with more utilization of homeless programs and emergency department care, and higher total health care costs increased.

**Meaning:**

These findings suggest that MMUs reach patients with greater needs and increase access to primary care and mental health services at higher costs.

## Teaser

This cohort study investigated the implementation of mobile medical unit care vs traditional care for veterans experiencing homelessness and assessed the associated costs and utilization of services.

## Introduction

Persons experiencing homelessness have a higher risk of poor health outcomes than the general population and face barriers to accessing regular medical care such as competing priorities, perceived stigma from health care professionals, lack of transportation to clinics, and difficulty making appointments for services.^1,2,3,4^ Consequently, this population experiences higher rates of delayed care and unmet needs for care than the general population.^5^

The Veterans Affairs (VA) health care system is the largest provider of institutional care to persons experiencing homelessness in the US (Peter H. Dougherty, BA. Testimony to the US Senate Committee on Veterans' Affairs, March 16, 2006). The VA system provides eligible veterans with comprehensive health care services and various supports to overcome barriers to care. More than 60 VA medical centers provide care to veterans experiencing homelessness through a patient-centered medical home model, the Homeless Patient Aligned Care Teams (HPACT) program, including more tailored care than usual primary care.^6,7^ The HPACT program consists of specialized medical care teams, often embedded with other homeless programs, that maintain small panels, provide interdisciplinary care, offer flexible scheduling, conduct community outreach, and provide services such as food assistance and transportation to improve access and use of VA primary care.

To conduct more extensive outreach to veterans less engaged in VA care and provide care that is more accessible, convenient, and culturally sensitive to those experiencing barriers to care in clinics and hospitals, the national HPACT program began a new mobile medical unit (MMU) program to provide health services in outfitted vans and other large vehicles to patients in community settings. MMU care was intended to supplement traditional primary care. A total of 24 VA medical centers received MMU vehicles outfitted with basic medical equipment to on a voluntary, rolling basis from October 1, 2023, to April 30, 2024.^8^

MMUs have been implemented within and outside the VA to reach populations experiencing access barriers; many of them provide primary care and population-based screening.^9,10,11,12,13^ MMUs have the potential to provide more timely care, since patients might otherwise delay or forgo care, leading to poorly managed conditions and unnecessary use of acute care. However, few rigorous studies to date have examined the effectiveness of MMUs.^14^ One study of patients in Massachusetts using an urban MMU providing health screenings found that patients experienced lower blood pressure and had fewer emergency department (ED) visits after visiting the MMU, amounting to $1.4 million in savings during 3 years.^13^ However, the study did not include a control group and did not focus on people experiencing homelessness, so the effects of MMUs on utilization and costs compared with traditional care in this population are unknown.

In this study, we examined the outcomes associated with early implementation of primary care MMUs through the HPACT program. We estimated changes in utilization for different types of VA outpatient care, different types of VA inpatient care, and VA health care costs before and after MMU implementation. We compared outcomes among veterans experiencing homelessness who used MMU care vs those who accessed traditional care. We hypothesized that MMU implementation would be associated with increased access to primary and mental health care and reduced avoidable inpatient care, with equivocal associations with total health care costs.

## Methods

This cohort study was conducted for quality improvement in partnership with the VA Homeless Programs Office as a quality improvement, nonresearch activity, so institutional review board approval and informed consent were deemed not necessary. We followed Strengthening the Reporting of Observational Studies in Epidemiology (STROBE) reporting guideline.

### MMU Implementation

Implementation of the HPACT MMU program was previously described in detail.^8,15^ In brief, 24 VA medical centers began MMU implementation between October 1, 2023, and April 30, 2024. While there was some variation, MMUs were staffed by interdisciplinary HPACT teams, including primary care clinicians, social workers, registered nurses or licensed vocational nurses, and other medical staff. There were no requirements for service availability, but sites provided similar primary care services, including health screenings and education, medication prescribing and administration, vaccinations, and a few other services. MMUs primarily visited VA and community housing sites, homeless shelters, and other urban community locations semiweekly or weekly. For more comprehensive care, HPACT teams could refer patients to VA clinics and hospitals. Any veteran enrolled in VA health care was eligible for MMU care; however, MMUs relied mainly on VA homeless programs, HPACT clinicians, and community partners to refer patients.

### Study Design and Cohort and Data Sources

We conducted a longitudinal study using a cohort of veterans experiencing homelessness in the 24 MMU sites for fiscal years 2022 to mid-2025 (October 1, 2021, to March 30, 2025). The patient cohort included veterans from the VA Homeless Registry that identified all patients enrolled in VA health care with a prior V code of homelessness or unstable housing in utilization records or who used VA homeless program services.^16^ In sensitivity analyses we (1) limited patients to a propensity score–matched sample and (2) compared all veterans experiencing homelessness in 24 MMU sites and 32 HPACT sites that did not implement MMUs (eFigure 1 in Supplement 1).

Data sources included VA administrative data on patient demographic characteristics from the VA Observational Medical Outcomes Partnership data, patients’ health care utilization and costs from the Corporate Data Warehouse inpatient and outpatient data, and Managerial Cost Accounting data, at least 2 years prior to and as long as 1.5 years following MMU implementation in MMU sites. Outcomes were measured in each fiscal quarter for patients’ utilization and costs.

### Study Measures

Our dependent variables included specific utilization and cost measures. Outpatient care was categorized by clinic location codes into primary care, specialty care, mental health care, substance use disorder (SUD) care, mental health intensive case management (MHICM), homeless programs, social work, and ED care. Inpatient care was categorized into medical or surgical, psychiatric, SUD, and domiciliary (residential rehabilitation treatment for mental health). We also measured VA outpatient, inpatient, and total costs of care from cost data that estimate the VA production costs of care.

Our key independent variable was site-specific MMU implementation. MMU implementation date was measured as the first fiscal quarter in which MMUs provided care to patients and documented through a designated program code in outpatient records. We considered intervention patients as those with at least 1 MMU encounter, and we considered usual care patients as those with at least 1 VA primary care visit in either regular primary care or homeless-tailored primary care but no MMU care during fiscal years 2024 through 2025.

For descriptive purposes and regression adjustment, we measured patients’ demographic characteristics, including age, sex, race and ethnicity, marital status, VA enrollment priority group (based on service-connected disability rating and VA means test), and rurality. Race and ethnicity were included because these factors influence the amount of VA care used by patients. We also measured 29 specific conditions (arthritis, asthma, cancer, chronic obstructive pulmonary disorder, type 2 diabetes, headache, heart disease, heart failure, hepatitis C virus infection, hypertension, low back pain, peripheral vascular disorder, pneumonia, prostatic hyperplasia, kidney disease, stroke, anxiety disorder, bipolar disorder, depression, mood disorders, posttraumatic stress disorder, schizophrenia, serious mental illness, and SUDs for alcohol, opioids, cannabis, stimulants, nicotine, and other substances) and Elixhauser comorbidity score based on diagnosis codes from all VA inpatient and outpatient encounter records in each year.^17^

### Statistical Analysis

The unit of analysis was the patient-quarter. Our primary analysis compared veterans experiencing homelessness who used MMU care vs those who accessed usual primary care only. In a descriptive analysis, we summarized the mean value or the proportion of patients with a given characteristic for MMU and comparison groups using standardized mean differences (SMD). An SMD of 0.20 was considered a meaningful difference.^18^

Using quasi-experimental methods, we conducted difference-in-differences (DID) analyses using linear 2-way fixed-effects models comparing outcomes between patient groups before and after MMU implementation with fixed effects for patient and fiscal quarter. Due to some outcomes not following a normal distribution, we also tested 2-part models. We also conducted event study models comparing differences in outcomes in each fiscal quarter relative to MMU implementation. After adjusting for trends over time common in both groups, the difference between the intervention and comparison groups after program implementation is considered the causal effect of the program. In both analyses, we adjusted for patients’ time-varying factors (VA priority group, Elixhauser comorbidity score, condition indicators) and adjusted SEs for clustering within sites. We examined parallel trends in outcomes prior to program implementation by comparing adjusted differences in each fiscal quarter and in each fiscal quarter relative to MMU implementation both visually and in regression models.

We conducted 2 sets of sensitivity analyses. First, we matched patients in the MMU and usual care groups using propensity scores with nearest neighbor matching based on demographic characteristics, mean Elixhauser comorbidity score, and mean utilization of the ED and homeless programs in the years prior to MMU implementation to compare higher-need veterans experiencing homelessness with those using MMU care. Second, we compared outcomes among all veterans experiencing homelessness in the 24 sites with MMUs and all veterans experiencing homelessness in 32 HPACT sites that did not implement MMUs, since any veteran experiencing homelessness in an MMU site could theoretically access MMU care. In both sensitivity analyses, we performed DID analysis with 2-way fixed effects and event study linear models comparing outcomes for the intervention and usual care groups before MMU and after MMU implementation similar to our primary analysis.

Statistical significance was indicated for testing whether the coefficients of fiscal-quarter indicators in the postimplementation period were different from 0 with a significance level of 2-sided *P* = .05. All analyses were conducted in Stata, version 18 (StataCorp LLC).

## Results

### Characteristics of MMU and Usual Care Groups

A total of 2700 veterans experiencing homelessness used MMU care. The mean (SD) age was 60.5 (12.6) years; 177 patients (7%) were female and 2523 (93%) were male. The mean (SD) Elixhauser comorbidity score was 3.3 (2.8). Compared with 131 991 veterans experiencing homelessness receiving usual primary care (mean [SD] age, 53.6 [15.6] years; 20 597 [16%] female, 111 394 [84%] male), patients in the MMU group were older and more likely to be male, not currently married, and eligible for VA care through the means test or Medicaid eligibility and Mexican Border or Gulf War eligibility than the comparison group. Patients in the MMU group had notably lower rates of posttraumatic stress disorder and higher rates of cocaine and nicotine use (all SMD>0.20). Prior to implementation of MMUs, patients in the MMU group had higher mean utilization per quarter of VA homeless programs (SMD = −0.33). Complete cohort characteristics are provided in Table 1.

**Table 1.** Characteristics of Patients Receiving Usual Care and MMU Care at Baseline^a^

Characteristic	Patient group		SMD
	Usual care (n = 131 864)^b^	MMU (n = 2700)^c^	
Age, mean (SD), y	53.6 (15.6)	60.5 (12.6)	−0.49
Sex, No. (%)			
Female	20 470 (16)	177 (7)	0.29
Male	111 394 (84)	2523 (93)	−0.29
Race and ethnicity, No. (%)			
Black	51 251 (39)	1132 (42)	−0.06
Hispanic	10 678 (8)	163 (6)	0.08
White	57 655 (44)	1120 (41)	0.05
Other race^d^	4119 (3)	85 (3)	−0.00
Unknown or missing	8161 (6)	200 (7)	−0.05
Marital status, No. (%)			
Divorced, separated, or widowed	58 195 (44)	1361 (50)	−0.13
Married	31 561 (24)	378 (14)	0.26
Single	40 568 (31)	930 (34)	−0.08
Unknown	1540 (1)	31 (1)	0.00
Priority group, No. (%)			
30% to 100% Service connected	64 111 (49)	705 (26)	0.48
10% to 20% Service connected, aid and attendance benefit	19 500 (15)	496 (18)	−0.10
Means test, Medicaid eligible; WWI, Mexican Border, or Gulf War	36 652 (28)	1201 (44)	−0.35
Not service connected, above means test	11 334 (9)	266 (10)	−0.04
Unknown	267 (0.2)	32 (1)	−0.12
Urban community, No. (%)	112 400 (85)	2446 (91)	−0.16
Elixhauser comorbidity score, mean (SD)^e^	2.9 (2.5)	3.3 (2.8)	−0.14
Medical conditions, No. (%)			
All cancers	9193 (8)	165 (7)	0.01
Arthritis	16 492 (14)	374 (16)	−0.08
Asthma	6082 (5)	92 (4)	0.05
Chronic obstructive pulmonary disease	12 557 (10)	364 (16)	−0.17
Congestive heart failure	7373 (6)	158 (7)	−0.03
Type 2 diabetes	25 889 (21)	546 (24)	−0.06
Headache	15 373 (13)	189 (8)	0.14
Hepatitis C virus infection	3659 (3)	142 (6)	−0.14
Hypertension	51 409 (43)	1133 (50)	−0.15
Ischemic heart disease	10 841 (9)	234 (10)	−0.04
Lower back pain	38 626 (32)	620 (27)	0.10
Peripheral vascular disease	5803 (5)	142 (6)	−0.06
Pneumonia	2399 (2)	79 (3)	−0.09
Prostatic hyperplasia	11 878 (10)	297 (13)	−0.10
Kidney failure	8565 (7)	194 (9)	−0.05
Stroke	6442 (5)	150 (7)	−0.05
Mental health conditions, No. (%)			
Serious mental illness	17 614 (15)	374 (16)	−0.05
Anxiety	35 646 (29)	490 (22)	0.18
Bipolar or manic disorder	10 187 (8)	180 (8)	0.02
Major depression	18 903 (16)	353 (16)	0.00
Other mood disorder	8713 (7)	157 (7)	0.01
Posttraumatic stress disorder	41 902 (35)	552 (24)	0.23
Schizophrenia related	9266 (8)	233 (10)	−0.09
SUD conditions, No. (%)			
Alcohol	29 336 (24)	674 (30)	−0.12
Opioid	6584 (5)	175 (8)	−0.09
Cannabis	15 360 (13)	334 (15)	−0.06
Cocaine	10 705 (9)	383 (17)	−0.24
Stimulant	8196 (7)	284 (12)	−0.19
Nicotine	20 728 (17)	576 (25)	−0.20
Other substances	7245 (6)	207 (9)	−0.12
No. of VA inpatient stays, per quarter, mean (SD)			
Medical or surgical	0.04 (0.3)	0.07 (0.3)	−0.09
Psychiatric	0.03 (0.2)	0.04 (0.3)	−0.06
Observation	0.02 (0.2)	0.04 (0.3)	−0.08
SUD	0.00 (0.1)	0.01 (0.1)	−0.03
Domiciliary	0.01 (0.1)	0.01 (0.1)	−0.03
No. of VA outpatient visits per quarter, mean (SD)			
Primary care	1.0 (1.6)	1.0 (1.6)	0.04
Mental health	1.0 (2.8)	0.8 (2.8)	0.05
Homeless programs	1.0 (2.8)	2.2 (4.1)	−0.33
Mental health intensive case management	0.1 (1.0)	0.1 (1.5)	−0.04
Substance use disorder	0.4 (2.3)	0.4 (2.4)	−0.03
Specialty care	0.8 (2.0)	0.8 (2.1)	0.01
Social work	<0.1 (0.3)	<0.1 (0.3)	−0.00
Emergency department	0.3 (0.8)	0.4 (1.3)	−0.16
VA health care costs per quarter, mean (SD)	$7854 ($23 662)	$10 121 ($26 457)	−0.09

Abbreviations: MMU, mobile medical unit; SMD, standardized mean difference; SUD, substance use disorder; VA, Department of Veterans Affairs.

^a^ All cohort patients were identified from VA medical centers that implemented MMUs and were in the VA homeless registry in fiscal year 2023.

^b^ Includes veterans experiencing homelessness who had at least 1 VA primary care visit but no MMU care for the fiscal years 2024 to 2025. Seven patients did not have any VA utilization in fiscal year 2023 and had missing values that year.

^c^ Includes veterans experiencing homelessness who had at least 1 MMU encounter.

^d^ Includes Asian Pacific Islander, Alaska Native, and Native Hawaiian.

^e^ Scores range from 0 to 22, with higher scores indicating more comorbidities.

**Figure 1.**
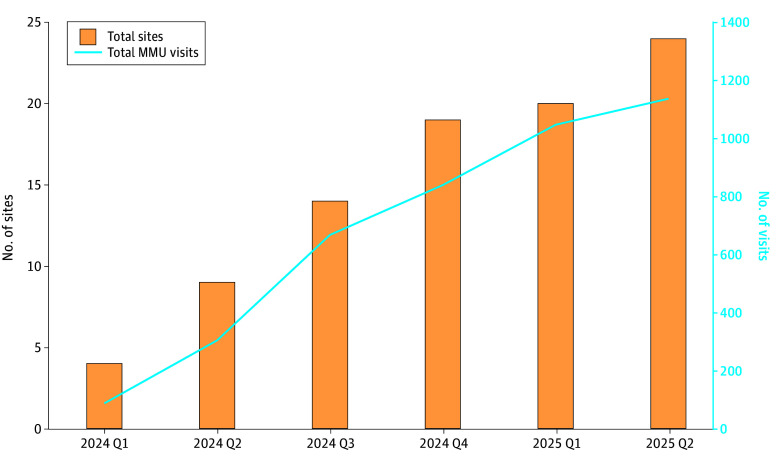
Number of Homeless Patient Aligned Care Teams Mobile Medical Unit (MMU) Sites and Visits Data are for fiscal years 2024, quarter 1 (Q1), to 2025, Q2.

In quarter 1 of 2024, 4 sites implemented MMUs and provided 94 MMU visits, and that increased to 24 sites providing 1236 MMU visits by quarter 2 of 2025 (Figure 1). Patients with any MMU use had a mean (SD) of 0.38 (0.71) MMU visits per quarter.

### Comparison of Time Trends in Outcomes in MMU and Usual Care Groups

Time trends in adjusted mean primary care visits, MHICM, and inpatient costs were similar between patients in the MMU and usual care groups prior to fiscal year 2024 (eFigure 2 in Supplement 1). For ED visits, homeless program visits, and inpatient medical or surgical care, the level of utilization was higher among patients using MMU, and it increased in the quarters prior to fiscal year 2024 relative to patients receiving usual care, so causality from MMU implementation cannot be inferred for these outcomes.

### DID and Event Study Results

In adjusted DID models, patients in the MMU group had a significantly greater mean number of visits per quarter for primary care (0.43; 95% CI, 0.30-0.56), homeless programs (1.40; 95% CI, 1.05-1.76), ED (0.06; 95% CI, 0.03-0.10), and MHICM (0.03; 95% CI, 0.01-0.06) and significantly fewer mean inpatient stays per quarter for inpatient SUD care (−0.002; 95% CI, −0.004 to −0.0004) and domiciliary care (−0.002; 95% CI, −0.004 to −0.001) after MMU implementation compared with usual care (Table 2). There were no differences in other types of care. Overall, patients in the MMU group had higher mean outpatient costs ($2243; 95% CI, $1854-$2632), lower mean inpatient costs (−$519; 95% CI, −$1030 to −$7), and higher mean total costs ($1724; 95% CI, $1172-$2276) per quarter after MMU implementation compared with usual care.

**Table 2.** Difference-in-Differences Estimates of Effects of Homeless Patient Aligned Care Teams MMU Implementation on Patients’ Utilization and Costs per Quarter^a^

Outcome	Difference in outcome per patient per quarter, mean (95% CI)	Difference in outcome, %^b^	*P* value for difference
Outpatient visits, No.			
Primary care	0.43 (0.30 to 0.56)	43	<.001
Specialty care	0.02 (−0.05 to 0.09)	3	.54
Emergency department	0.06 (0.03 to 0.10)	15	<.001
Homeless programs	1.40 (1.05 to 1.76)	64	<.001
Mental health care	0.04 (−0.11 to 0.02)	5	.61
Substance use disorder care	0.003 (−0.08 to 0.09)	0	.95
Social work	0.02 (−0.01 to 0.04)	58	.28
Mental health intensive case management	0.03 (0.01 to 0.06)	30	.03
Inpatient stays, No.			
Medical or surgical	0.001 (−0.01 to 0.01)	1	.75
Psychiatric	0.001 (−0.01 to 0.01)	3	.77
SUD treatment	−0.002 (−0.004 to −0.0004)	20	.02
Domiciliary	−0.002 (−0.004 to −0.001)	20	.02
VA health care costs, $			
Total outpatient costs	2243 (1854 to 2632)	40	<.001
Total inpatient costs	−519 (−1030 to −7)	−12	.06
Total costs	1724 (1172 to 2276)	17	<.001

Abbreviations: MMU, mobile medical unit; SUD, substance use disorder; VA, Veterans Affairs.

^a^ Difference in differences estimates were obtained from linear 2-way fixed-effects models with MMU implementation as the primary independent variable and comparing patients using MMUs with those using usual care, adjusting for patient and fiscal year quarter fixed effects, patient age, VA enrollment priority group, Elixhauser comorbidity score, and 29 specific health conditions with SEs adjusted for VA sites. MMU implementation date varied by site.

^b^ Calculated from mean baseline values in fiscal year 2023.

**Figure 2.**
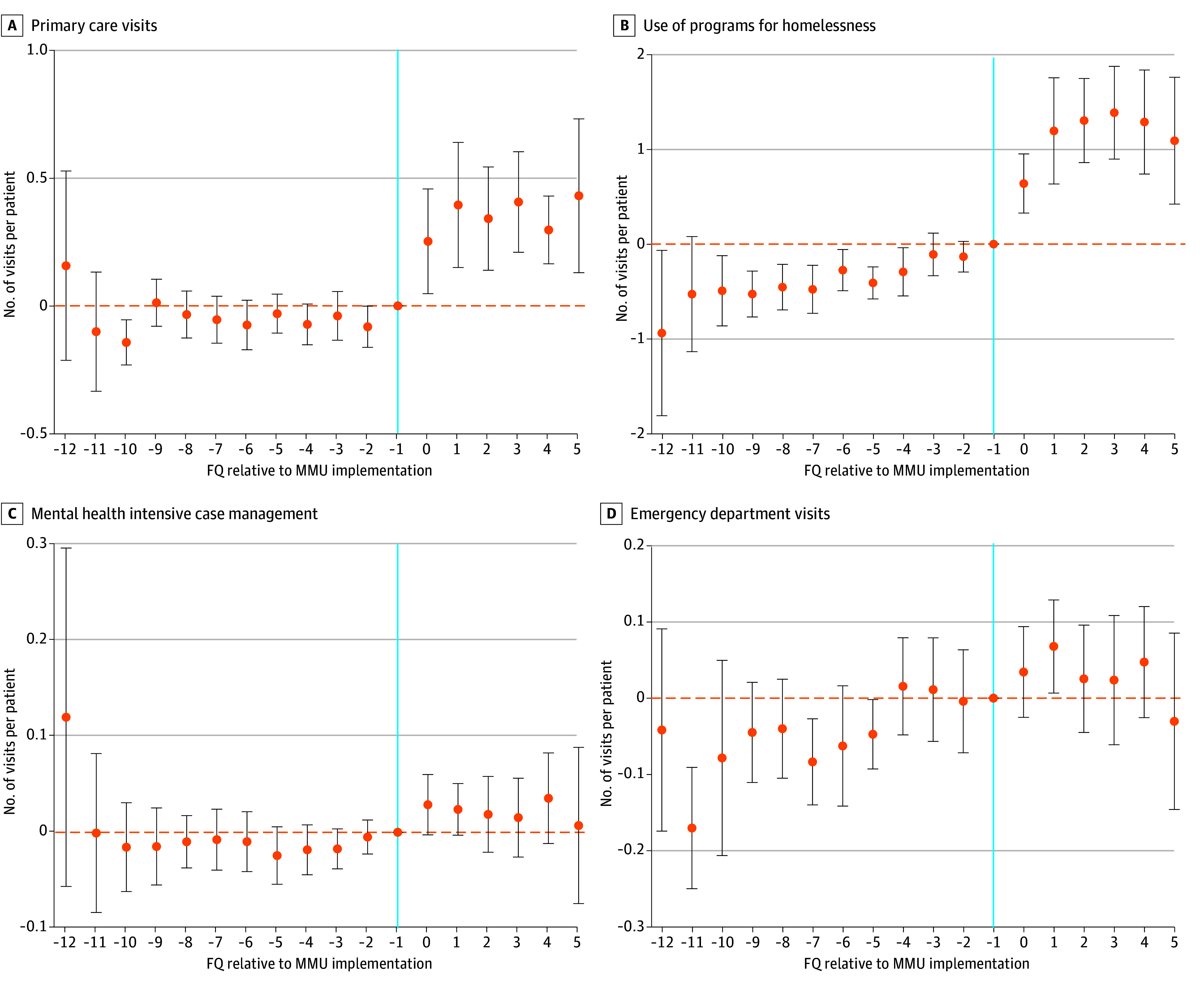
Event Study Estimates of Differences in Outpatient Utilization for Patients Using Mobile Medical Units (MMUs) and Usual Care Before and After MMU Implementation Vertical blue line marks the fiscal quarter (FQ) prior to MMU implementation. Horizontal, yellow dashed lines mark 0, or no difference between MMU and comparison groups. Event study estimates were obtained from linear models with time to MMU implementation as the primary independent variable and comparing patients using MMU with those receiving usual care, adjusting for patient and FQ fixed effects, patient age, Veterans Affairs (VA) enrollment priority group, Elixhauser comorbidity score, and 29 specific health conditions with SEs adjusted for VA sites. MMU implementation date varied by site. Data points indicate point estimates; error bars indicate 95% CIs.

**Figure 3.**
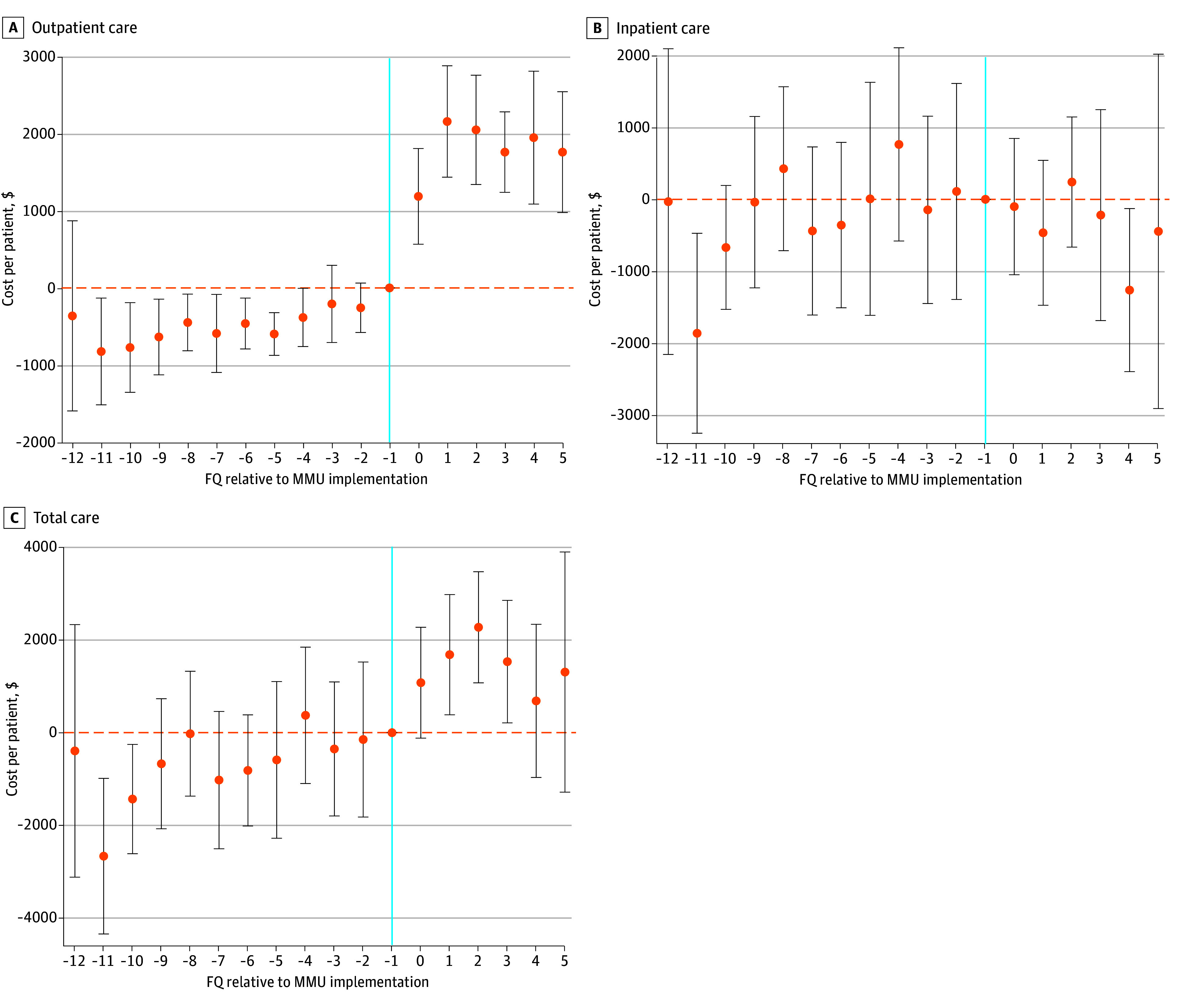
Event Study Estimates of Differences in Mean Veterans Affairs (VA) Health Care Costs for Patients Using Mobile Medical Units (MMUs) and Usual Care Before and After MMU Implementation Vertical blue line marks the fiscal quarter (FQ) prior to MMU implementation. Horizontal, yellow dashed lines mark 0, or no difference between MMU and comparison groups. Event study estimates were obtained from linear models with time to MMU implementation as the primary independent variable and comparing patients using MMU with those using usual care, adjusting for patient and FQ fixed effects, patient age, VA enrollment priority group, Elixhauser comorbidity score, and 29 specific health conditions with SEs adjusted for VA sites. MMU implementation date varied by site. Data points indicate point estimates; error bars indicate 95% CIs.

In adjusted DID analyses with 2-part models, some outcomes had smaller differences, although lack of patient fixed effects could have increased bias (eTable 1 in Supplement 1). In event study analysis, adjusted differences in mean outpatient (Figure 2 and eFigure 3 in Supplement 1) and inpatient utilization (eFigure 4 in the Supplement) and costs (Figure 3) that were significantly different among patients in the MMU group in DID models occurred in the first quarter after MMU implementation; this trend continued in subsequent quarters, although differences in inpatient care were not consistently lower in postimplementation quarters. Prior to MMU implementation, there were few differences in adjusted mean outcomes and trends between groups. However, there was lower utilization of ED and homeless programs (Figure 2) and lower costs of outpatient care (Figure 3) that trended upward for the MMU group before implementation, so residual differences between groups could not be fully adjusted for in our models. ED and homeless program utilization and outpatient costs increased in the MMU group immediately after MMU implementation, suggesting some association with these outcomes and MMU implementation.

### Sensitivity Analyses

We matched patients in the usual care group (n = 2217) with those in the MMU group (n = 2277) with balanced characteristics between groups (eTable 2 in Supplement 1). Outpatient care results for event study models were similar to those in the primary analysis except for no differences in MHICM care and increased SUD visits for patients in the MMU group (eFigure 5 in Supplement 1). There were increases in most inpatient care types and higher inpatient and total costs for the MMU group after MMU implementation. However, event study graphs indicated different levels and patterns for primary care, homeless programs, and ED care between groups prior to MMU implementation, suggesting unobserved time-varying factors affected these outcomes.

In comparisons of all veterans experiencing homelessness in MMU sites (n = 173 488) and all veterans experiencing homelessness in other HPACT sites (n = 154 581), there were no significant differences in outcomes in DID models and event study models. There was a small increase in mean homeless program visits in MMU sites following MMU implementation (eFigure 6 in Supplement 1).

## Discussion

This study is the first, to our knowledge, to investigate the implementation of a national MMU program that focuses on veterans experiencing homelessness and document its association with health services utilization and costs. Although only 1.6% of veterans experiencing homelessness obtained MMU care in the first 18 months of the program, MMUs provided care to veterans at higher risk who used more homeless program services and had higher comorbidity levels and more SUD conditions. Implementation of MMUs was associated with small increases in primary care and MHICM care and decreases in inpatient SUD and domiciliary care. Patients in the MMU group also increased their total health care costs by more than $1700 per quarter after MMU implementation. While we found higher utilization of homeless programs and ED care among patients in the MMU group, we could not establish causal effects of the MMU program, only associations with these outcomes due to differences in preimplementation patterns.

Most of the increased utilization of primary care among patients in the MMU group appeared due to use of MMUs rather than referral to usual care. MMUs may have provided more convenient care and reduced travel barriers for some patients to supplement traditional clinic-based care. While we did not find increases in overall mental health care, we found small increases in utilization of MHICM care in the MMU group that may be appropriate for high-need patients who could benefit from more intensive mental health services. Greater utilization of homeless program services among patients receiving MMU care also suggests close partnerships between MMUs and housing and other programs to address homelessness, so it is important to track the effects of MMUs on veterans’ housing status in the future.

Since we found an association between use of ED care and MMU implementation, it is unclear whether MMUs can reduce ED care for patients with serious health problems that require more than basic services. While we found patients in the MMU group decreased use of inpatient SUD care and domiciliary care for mental health conditions, it is unknown whether MMUs directly affected this care through appropriate medication prescribing or timely referrals to specialty clinicians or whether MMUs increased enrollment in housing programs, which led to reduced inpatient care. Future work is needed to examine these issues in more detail.

Patients in the MMU group overall had increased outpatient and total health care costs relative to those receiving usual care. Due to the relatively small proportion of veterans experiencing homelessness who used MMU care, mean costs of all veterans experiencing homelessness in MMU sites did not significantly increase compared with HPACT sites that did not implement MMUs. Cost differences in this study may be underestimated because they did not include the cost of the MMU vehicles or maintenance and other expenses. Overall, more data on the value, or the cost per change in health outcome, from MMU implementation in the future can help justify the increased resource use in MMU sites.

In analysis of matched patients in the MMU and comparison groups, we found larger differences in preimplementation outcome trends than our primary analysis. Therefore, our data could not address unobserved time-varying differences such as dynamic changes in housing status or health status and precludes interpretation of causality of MMU implementation on some outcomes in the matched cohort. In comparisons of MMU sites and HPACT sites without MMUs, the lack of differences in outcomes may be partly due to the small proportion of veterans experiencing homelessness and using MMU care, as many sites were in early stages of their implementation. It will be important to track effects over a longer term as sites gain more experience with MMUs.

Our findings differ from those of prior studies of MMUs providing screening services to nonveterans, since we found more, not fewer, ED visits,^13,19^ although 1 study of MMUs providing services to persons experiencing homelessness found nonsignificantly higher ED and hospitalization utilization.^10^ MMUs outside the VA have been effective at engaging adults with SUDs, so reducing stigma around treatment and building trust may be key aspects of MMU care.^11,20^

### Limitations

This study had several limitations. We were not able to include VA-purchased community care or care not provided by the VA, so we may have undermeasured differences in outcomes between patients, especially if patients receiving MMU care obtain more care from community health professionals. We observed residual differences between patients using MMUs and patients using usual care in the primary analysis and propensity score–matched analysis for some outcomes prior to MMU implementation; therefore, we could not account for unmeasured time-varying factors affecting differences in outcomes between patient groups. Some of the sites had only implemented MMUs for 1 or 2 quarters, and there may be heterogeneous site effects, especially when some MMUs had more experience providing care. These findings may not be generalizable to MMUs outside of the VA health care system, since other systems may not provide comprehensive medical, social, and housing services with low patient cost-sharing.

## Conclusions

In this cohort study of veterans experiencing homelessness, we found that MMU implementation was associated with increased use of primary care and mental health services and decreased use of inpatient mental health and SUD care, so MMUs may be an effective modality to provide timely care for patients at higher risk. However, the increase in costs of providing care to patients using MMUs may temper expectations on immediate cost savings from implementing MMUs. Data on the effects of MMU on longer-term engagement in care and health and housing outcomes are ultimately needed to establish evidence on effectiveness and bolster efforts to disseminate MMUs on a larger scale.

## Notes

Supplement 1. **eFigure 1.** Flowchart of Study Samples **eFigure 2.** Patterns in Outcomes by Fiscal Quarter for Patients in MMU and Usual Care Groups, Adjusted by Patient Characteristics **eFigure 3.** Event Study Charts for Outpatient Visits for Specialty Care, Mental Health Care, Substance Use Disorder Care, and Social Work in Primary Analysis **eFigure 4.** Event Study Charts for Inpatient Stays for Medical or Surgical Care, Psychiatric Care, Substance Use Disorder Care, and Domiciliary in Primary Analysis **eTable 1.** Difference-in-Differences Estimates Using 2-Part Models **eFigure 5.** Event Study Charts for All Outcomes for Propensity Score–Matched Cohort of the MMU and Usual Care Groups **eTable 2.** Test of Bias Between Propensity Score–Matched Cohort of the MMU and Usual Care Groups, Before and After Matching **eFigure 6.** Event Study Charts for all Outcomes Comparing All Veterans Experiencing Homelessness in MMU Sites and Other HPACT Sites

Supplement 2.
Data Sharing Statement
