# Error in Byline

**DOI:** 10.1001/jamanetworkopen.2026.9718

**Published:** 2026-04-06

In the Original Investigation titled “Utilization and Costs of Mobile Medical Units for Veterans Experiencing Homelessness,”^1^ published January 30, 2026, author Jack Tsai was incorrectly assigned a middle initial in the byline. This article has been corrected.
