# Supplementary material for: Utilization and Costs of Mobile Medical Units for Veterans Experiencing Homelessness
Source: JAMA Netw Open. 2026 Jan 30;9(1):e2555068. doi: 10.1001/jamanetworkopen.2025.55068 (PMC12859722; doi:10.1001/jamanetworkopen.2025.55068)
Supplement: Supplement 1. — eFigure 1. Flowchart of Study Samples eFigure 2. Patterns in Outcomes by Fiscal Quarter for Patients in MMU and Usual Care Groups, Adjusted by Patient Characteristics eFigure 3. Event Study Charts for Outpatient Visits for Specialty Care, Mental Health Care, Substance Use Disorder Care, and Social Work in Primary Analysis eFigure 4. Event Study Charts for Inpatient Stays for Medical or Surgical Care, Psychiatric Care, Substance Use Disorder Care, and Domiciliary in Primary Analysis eTable 1. Difference-in-Differences Estimates Using 2-Part Models eFigure 5. Event Study Charts for All Outcomes for Propensity Score–Matched Cohort of the MMU and Usual Care Groups eTable 2. Test of Bias Between Propensity Score–Matched Cohort of the MMU and Usual Care Groups, Before and After Matching eFigure 6. Event Study Charts for all Outcomes Comparing All Veterans Experiencing Homelessness in MMU Sites and Other HPACT Sites [file jamanetwopen-e2555068-s001.pdf]

Supplementary Online Content

Yoon J, Chow A, Weber J, Wong E, Blonigen D, Tsai J. Utilization and costs of mobile medical units for veterans experiencing homelessness. *JAMA Netw Open.* 2026;9(1):e2555068. doi:10.1001/jamanetworkopen.2025.55068

eFigure 1. Flowchart of Study Samples

eFigure 2. Patterns in Outcomes by Fiscal Quarter for Patients in MMU and Usual Care Groups, Adjusted by Patient Characteristics

eFigure 3. Event Study Charts for Outpatient Visits for Specialty Care, Mental Health Care, Substance Use Disorder Care, and Social Work in Primary Analysis

eFigure 4. Event Study Charts for Inpatient Stays for Medical or Surgical Care, Psychiatric Care, Substance Use Disorder Care, and Domiciliary in Primary Analysis

eTable 1. Difference-in-Differences Estimates Using 2-Part Models

eFigure 5. Event Study Charts for All Outcomes for Propensity Score–Matched Cohort of the MMU and Usual Care Groups

eTable 2. Test of Bias Between Propensity Score–Matched Cohort of the MMU and Usual Care Groups, Before and After Matching

eFigure 6. Event Study Charts for all Outcomes Comparing All Veterans Experiencing Homelessness in MMU Sites and Other HPACT Sites

This supplementary material has been provided by the authors to give readers additional information about their work.

© 2026 Yoon J et al. *JAMA Network Open*.

eFigure 1. Flowchart of Study Samples

A. Primary cohort: mobile medical unit (MMU) patients and usual care patients in MMU sites

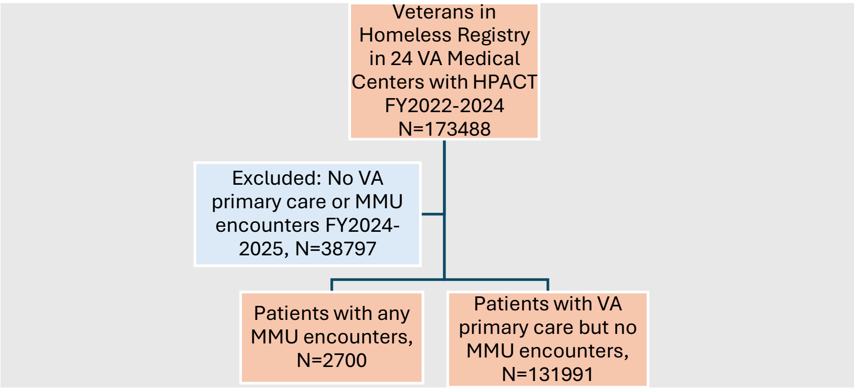

B. Sensitivity analysis: propensity-matched cohort of mobile medical unit patients and usual care patients in MMU sites

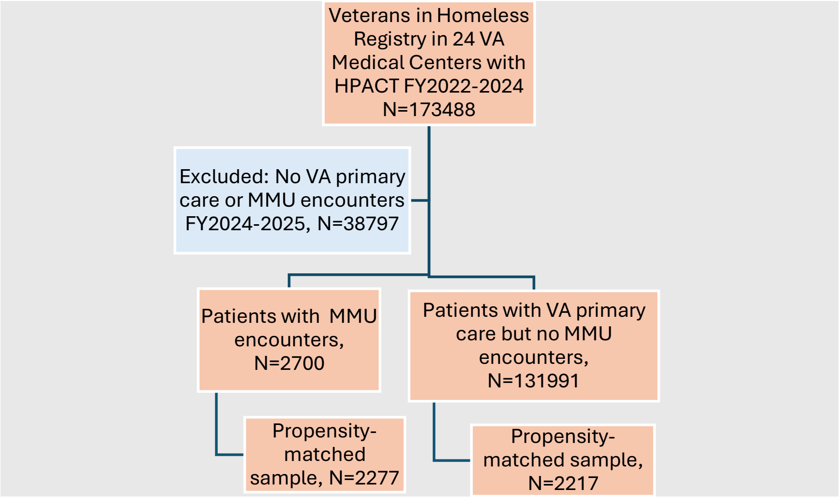

C. Sensitivity analysis: Veterans experiencing homelessness in mobile medical unit sites and other Homeless Patient Aligned Care Team sites

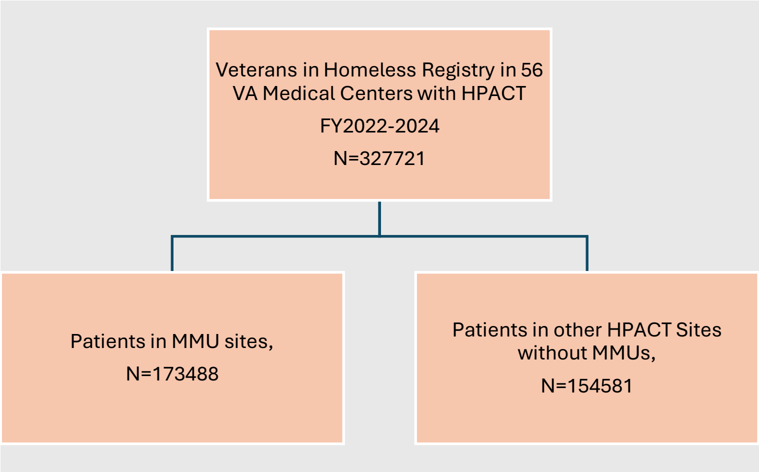

© 2026 Yoon J et al. *JAMA Network Open*.

eFigure 2. Patterns in Outcomes by Fiscal Quarter for Patients in MMU and Usual Care Groups, Adjusted by Patient Characteristics

A. Primary care, B. Homeless programs, C. Emergency department, D. Mental health intensive case management, E. Specialty care, F. Mental health, G. Substance use disorders, H. Social work, I. Inpatient medical/surgical, J. Inpatient psychiatric, K. Inpatient substance use disorders, L. Domiciliary, M. Outpatient costs, N. Inpatient costs, O. Total costs.

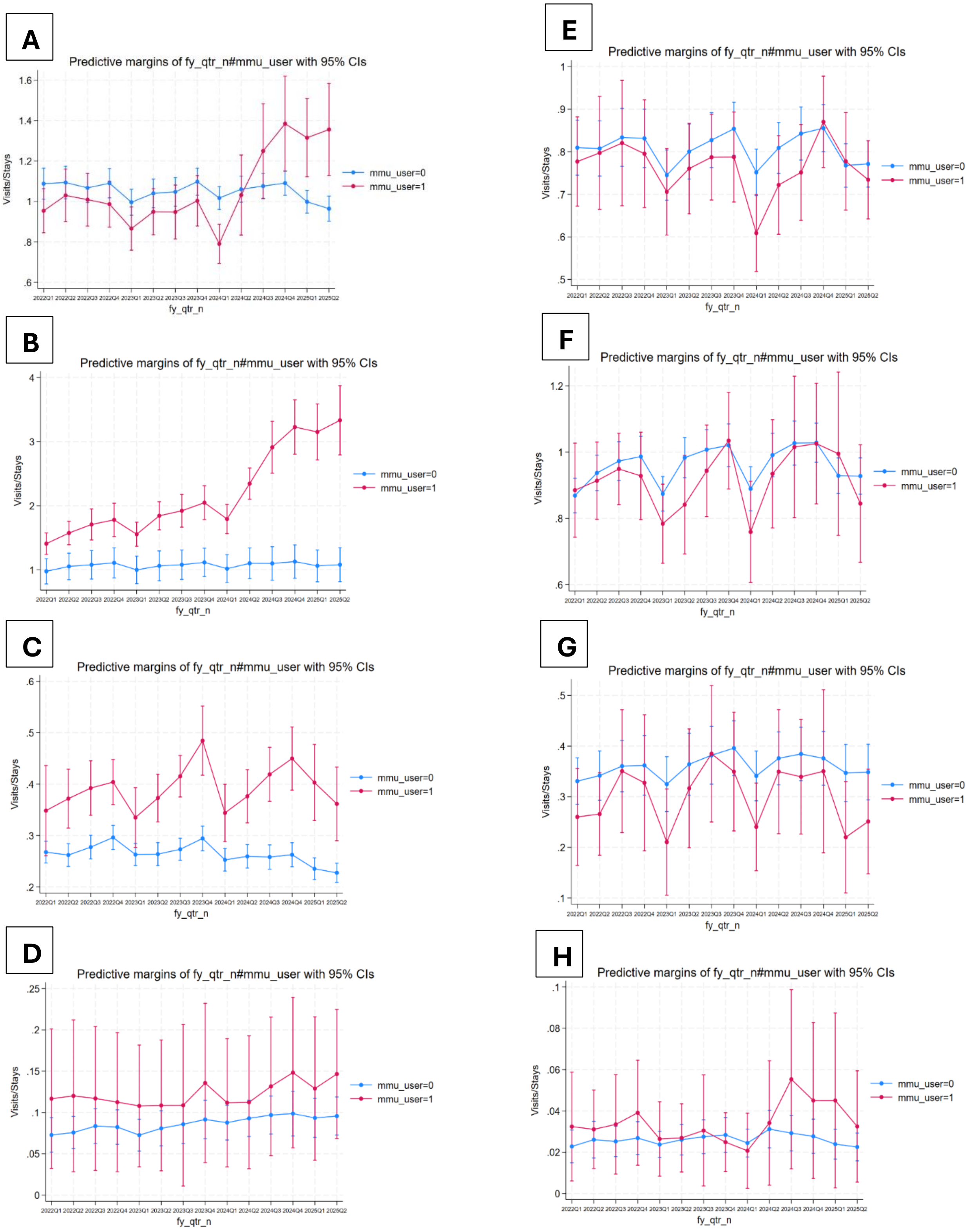

© 2026 Yoon J et al. *JAMA Network Open*.

I

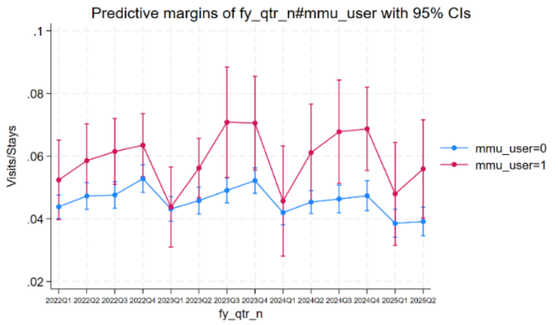

M

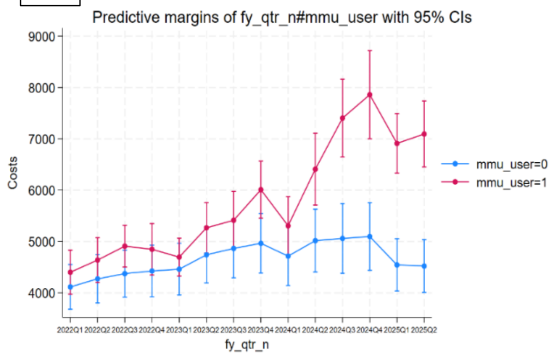

J

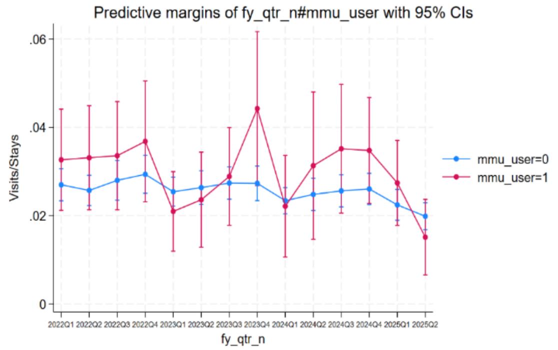

N

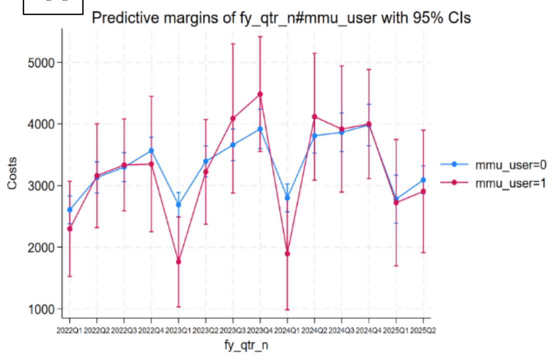

K

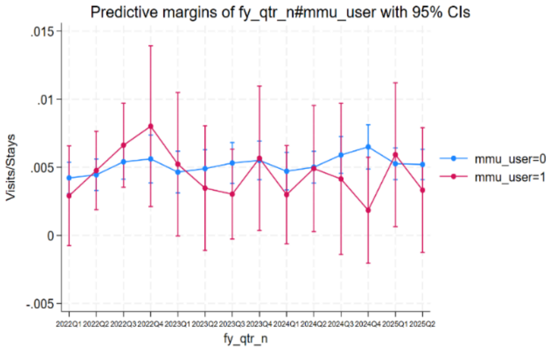

O

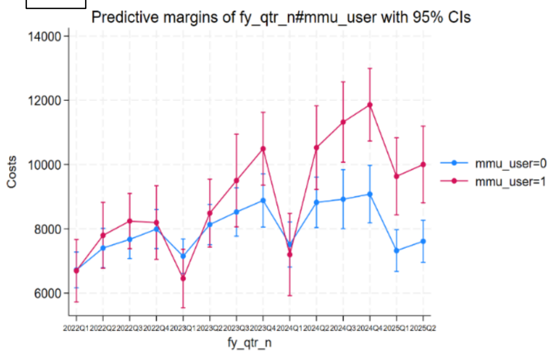

L

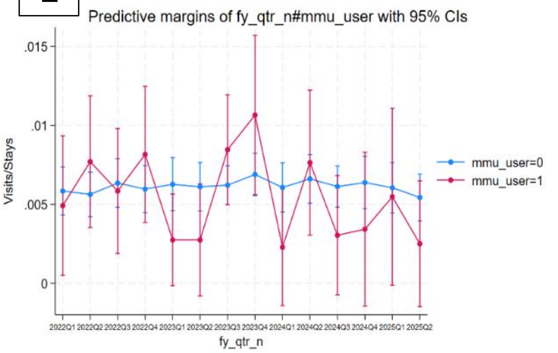

© 2026 Yoon J et al. *JAMA Network Open*.

eFigure 3. Event Study Charts for Outpatient Visits for Specialty Care, Mental Health Care, Substance Use Disorder Care, and Social Work in Primary Analysis

A. Specialty care, B. Mental health care, C. Substance use disorder care, D. Social work. Time = fiscal quarter relative to MMU implementation.

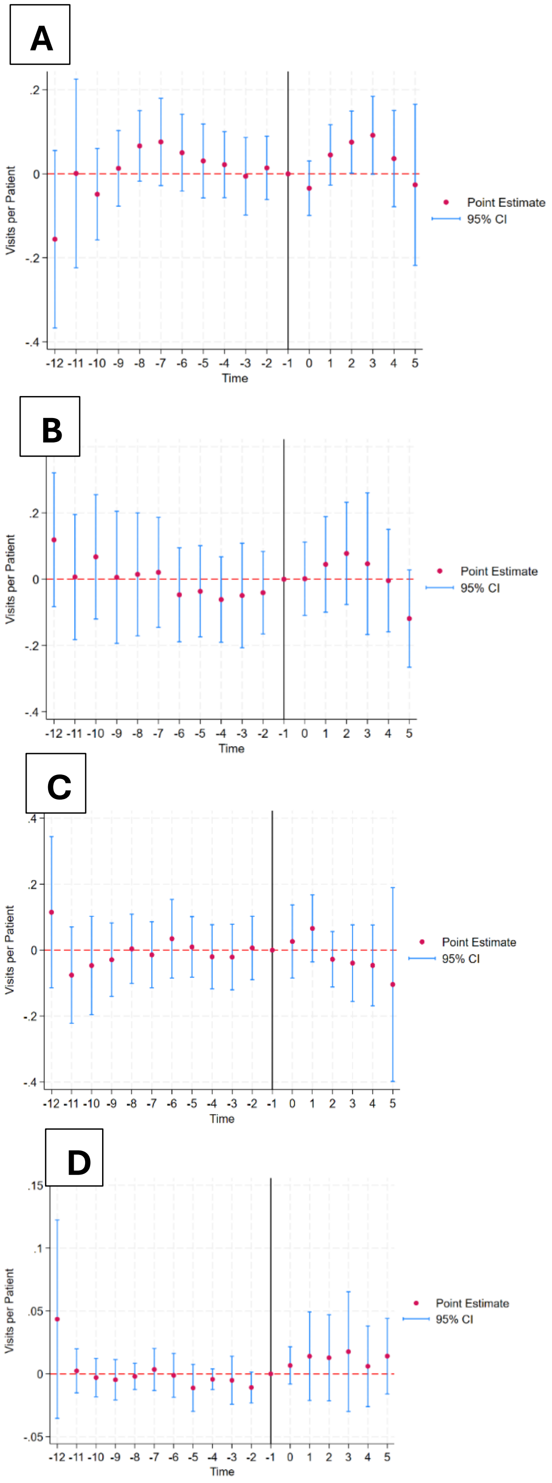

© 2026 Yoon J et al. *JAMA Network Open*.

eFigure 4. Event Study Charts for Inpatient Stays for Medical or Surgical Care, Psychiatric Care, Substance Use Disorder Care, and Domiciliary in Primary Analysis

A. Medical/surgical care, B. Psychiatric care, C. Substance use disorder care, D. Domiciliary. Time = fiscal quarter relative to MMU implementation.

A

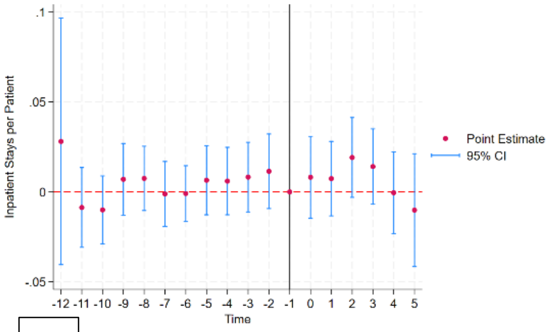

B

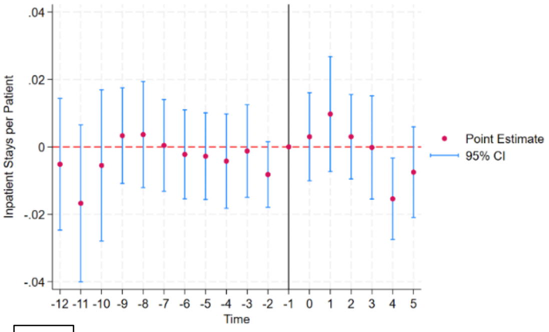

C

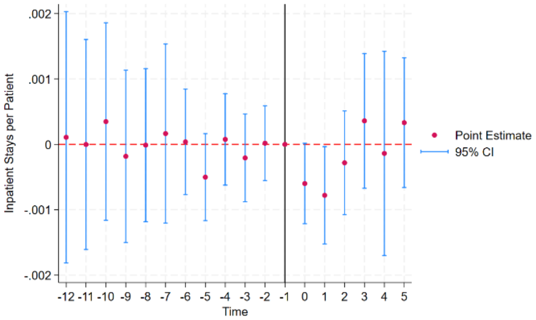

D

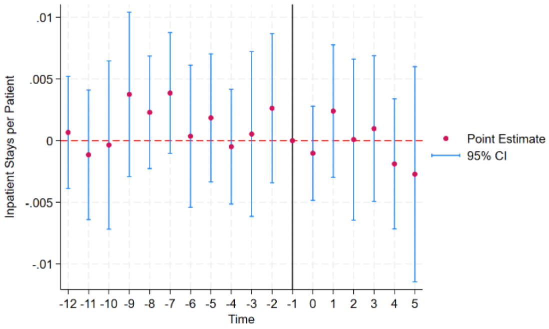

© 2026 Yoon J et al. *JAMA Network Open*.

eTable 1. Difference-in-Differences (DID) Using 2-Part Models[a]

Outcome	Mean Difference in Outcome per Patient per Quarter (DID Estimates)	Percent Difference in Outcome	P-Value for Difference
**Outpatient Visits**			
Primary Care	0.08 (-0.05, 0.21)	8%	0.21
Specialty Care	-0.07 (-0.13, -0.01)	-9%	0.02
Emergency Department	0.04 (0.01, 0.08)	10%	0.01
Homeless Programs	0.73 (0.51, 0.94)	33%	<0.01
Mental Health Care	-0.09 (-0.22, 0.04)	-11%	0.17
Substance Use Disorder Care	-0.03 (-0.09, 0.03)	-8%	0.32
Mental Health Intensive Case Management	0.03 (-0.02, 0.08)	30%	0.19
Social Work	0.003 (-0.01, 0.02)	12%	0.69
**Inpatient Stays**			
Medical/Surgical	0.000 (-0.01, 0.01)	0%	0.98
Psychiatric	-0.001 (-0.004, 0.002)	-3%	0.54
Substance Use Disorder Treatment	-0.002 (-0.003, -0.001)	-20%	0.003
Domiciliary	-0.002 (-0.003, -0.001)	-20%	<0.01
**VA Health Care Costs, in US Dollars**			
Total Outpatient Costs	$903 (416, 1390)	16%	<0.01
Total Inpatient Costs	-$953 (-1312, -593)	-21%	<0.01
Total Costs	$477 (-471, 1426)	5%	0.32

[a] DID estimates were obtained from two-part models using a logistic regression estimating any utilization or costs in the first part and a generalized linear regression with appropriate family distribution and link function estimating the amount of utilization or cost for those with any utilization or costs in the second part. In all models, MMU implementation was the primary independent variable, and MMU patients were compared with usual care patients, adjusting for fiscal-quarter fixed effects, patient age, VA enrollment priority group, Elixhauser comorbidity score, and 29 specific health conditions with standard errors adjusted for VA sites. MMU implementation date varied by site. Unlike models used in the primary analysis, these models did not include patient fixed effects.

© 2026 Yoon J et al. *JAMA Network Open*.

eFigure 5. Event Study Charts for All Outcomes for Propensity Score–Matched Cohort of the MMU and Usual Care Groups

A. Primary care, B. Homeless programs, C. Emergency Department, D. Mental health intensive case management, E. Specialty care, F. Mental health, G. Substance use disorder care. H. Social work, I. Inpatient medical/surgical, J. Inpatient psychiatric, K. Inpatient substance use disorder, L. Domiciliary, M. Outpatient costs, N. Inpatient costs, O. Total costs. Time = fiscal quarter relative to MMU implementation.

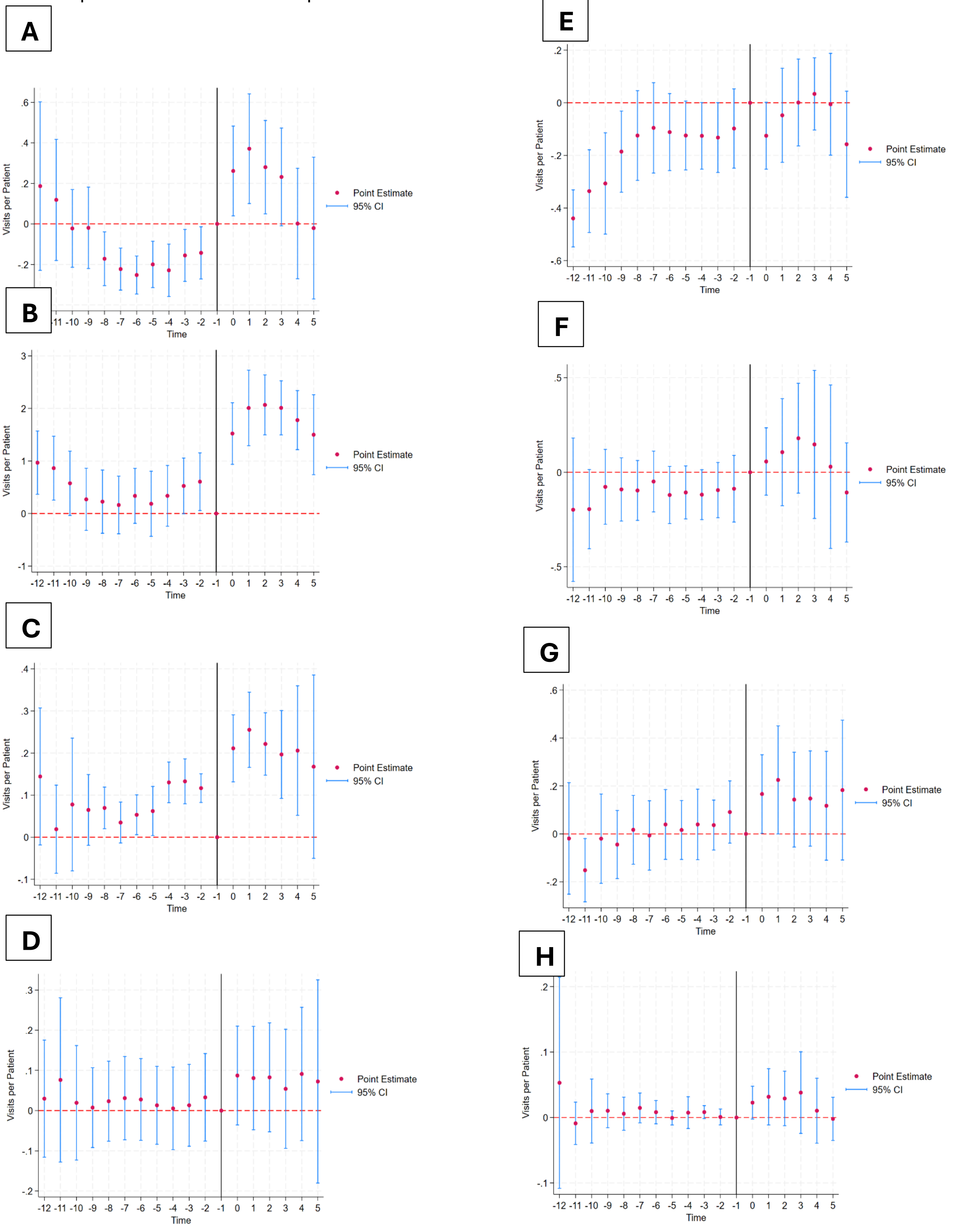

© 2026 Yoon J et al. *JAMA Network Open*.

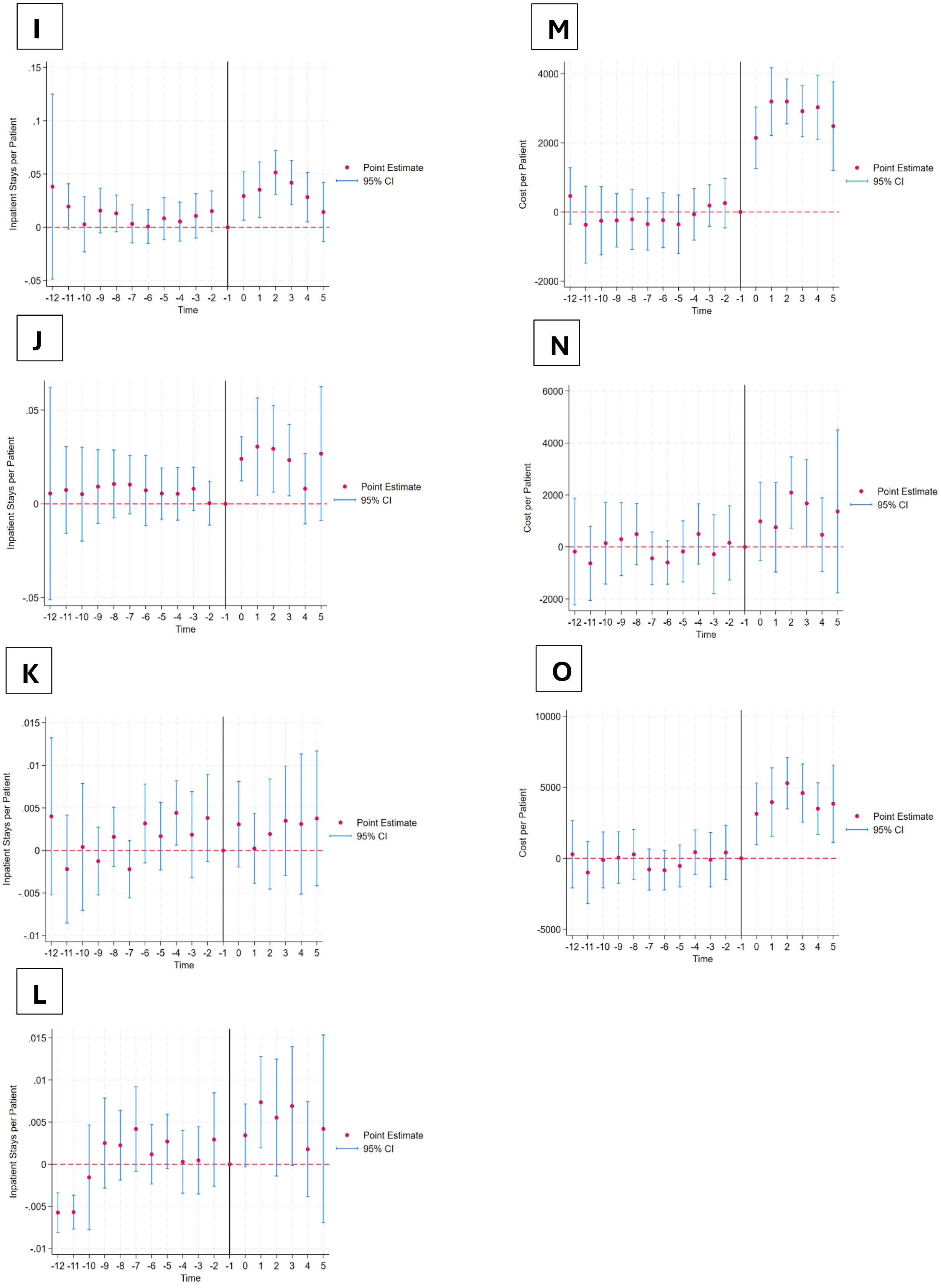

© 2026 Yoon J et al. *JAMA Network Open*.

eTable 2. Test of Bias Between Propensity Score–Matched Cohort of the MMU and Usual Care Groups, Before and After Matching

Patient characteristic		MMU Patients	Usual Care Patients	% Bias
		Mean/Frequency		
Age	Unmatched	60.8	53.9	48.5
	Matched	61.2	62.1	-6.8
30% to 100% service connected	Unmatched	0.27	0.49	-47.1
	Matched	0.29	0.29	-1.0
Means test/Medicaid eligible; WW1/Mexican Border/Gulf War	Unmatched	0.44	0.28	35.4
	Matched	0.43	0.44	-2.8
Single, never married	Unmatched	0.34	0.31	7.9
	Matched	0.35	0.35	-0.9
Elixhauser comorbidity score	Unmatched	3.50	2.99	18.9
	Matched	3.58	3.47	4.0
ED visits	Unmatched	0.22	0.12	17.2
	Matched	0.22	0.18	7.6
Homeless program visits	Unmatched	1.59	0.75	31.6
	Matched	1.59	1.39	7.7
Diagnosed with substance use disorder	Unmatched	0.51	0.39	23.5
	Matched	0.52	0.45	14.2

© 2026 Yoon J et al. *JAMA Network Open*.

eFigure 6. Event Study Charts for all Outcomes Comparing All Veterans Experiencing Homelessness in MMU Sites and Other HPACT Sites

A. Primary care, B. Homeless programs, C. Emergency department, D. Mental health intensive case management, E. Specialty care, F. Mental health care, G. Substance use disorder care, H. Social work, I. Inpatient medical/surgical, J. Inpatient psychiatric, K. Inpatient substance use disorders, L. Domiciliary, M. Outpatient costs, N. Inpatient costs, O. Total costs. Time = fiscal quarter relative to MMU implementation.

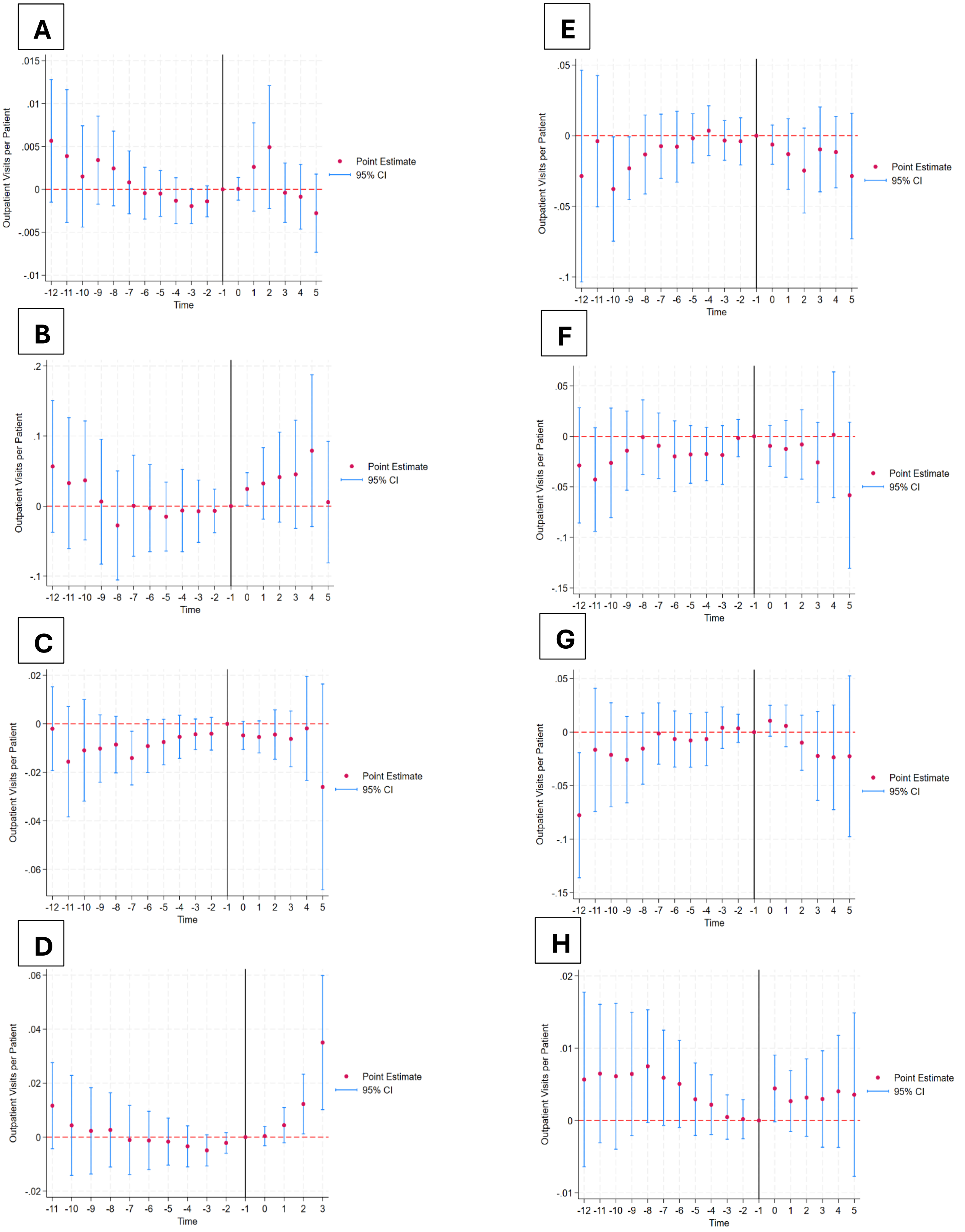

© 2026 Yoon J et al. *JAMA Network Open*.

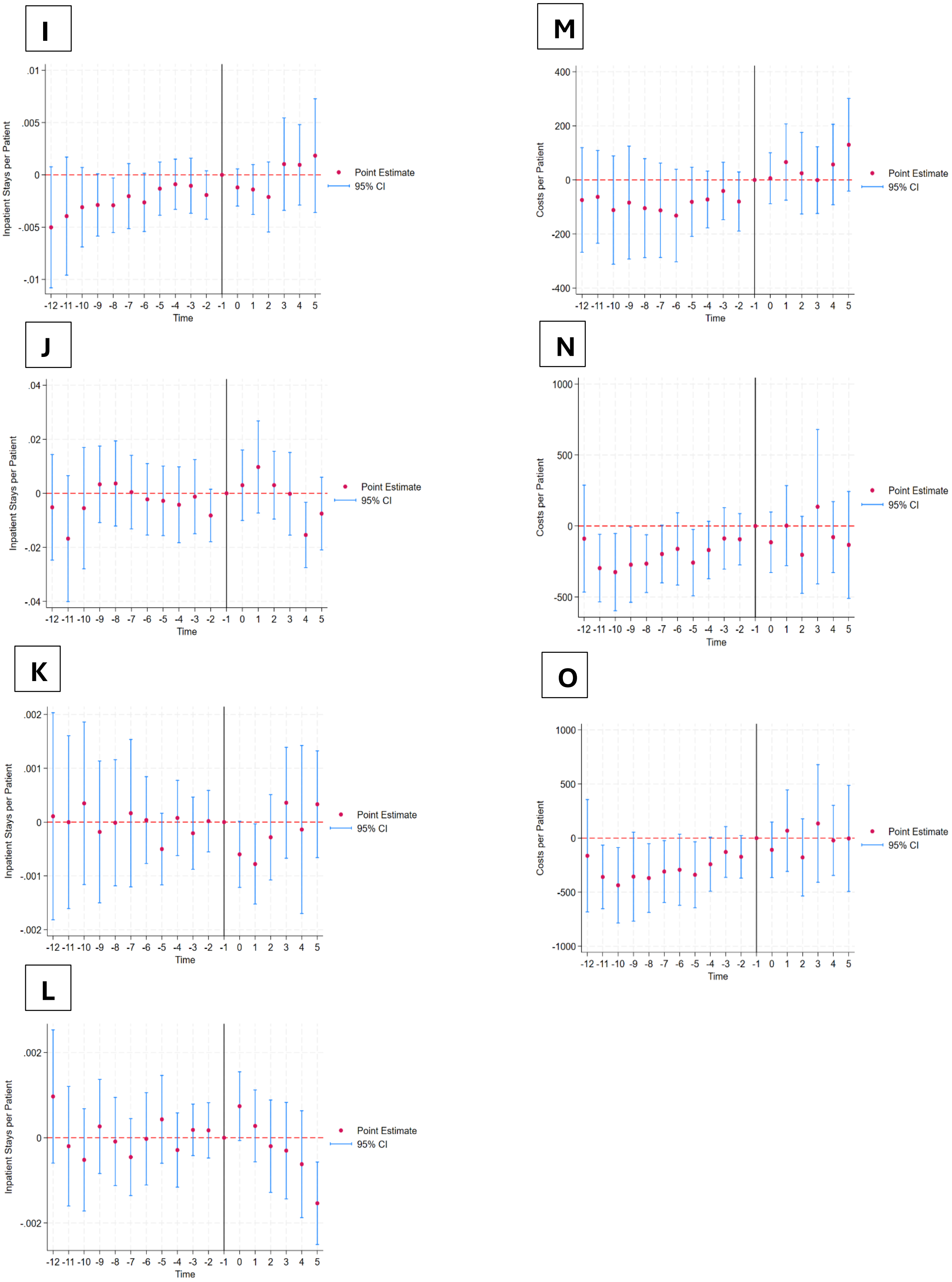

© 2026 Yoon J et al. *JAMA Network Open*.
