# Supplementary material for: Utilization and Costs of Mobile Medical Units for Veterans Experiencing Homelessness
Source: JAMA Netw Open. 2026 Jan 30;9(1):e2555068. doi: 10.1001/jamanetworkopen.2025.55068 (PMC12859722; doi:10.1001/jamanetworkopen.2025.55068)
Supplement: Supplement 2. — Data Sharing Statement [file jamanetwopen-e2555068-s002.pdf]

Data Sharing Statement

Yoon. Utilization and Costs of Mobile Medical Units for Veterans Experiencing Homelessness. *JAMA Netw Open*. Published January 29, 2026. doi:10.1001/jamanetworkopen.2025.55068

Data
Data available: No

Additional Information
Explanation for why data not available: Data were obtained from the VA National Center on Homelessness Among Veterans for the purposes of quality improvement for this study. The study team does not have authorization to share these data.
